# Occult neoplastic cells in lymph node sinuses and recurrence/metastasis of stage II/III gastric cancer

**DOI:** 10.3892/ol.2013.1660

**Published:** 2013-11-06

**Authors:** YASUTOMO SEKIDO, MASAYA MUKAI, MASASHI YAMAZAKI, TAKAYUKI TAJIMA, SOUICHIROU YAMAMOTO, SAYURI HASEGAWA, KYOKO KISHIMA, TAKUMA TAJIRI, NAOYA NAKAMURA

**Affiliations:** 1 Department of Pathology, Tokai University School of Medicine, Bohseidai, Isehara, Kanagawa 259-1193, Japan; 2 Department of Pathology, Isehara Kyodo Hospital, Isehara, Kanagawa 259-1132, Japan; 3 Department of Surgery, Tokai University Hachioji Hospital, Hachioji, Tokyo 192-0032, Japan; 4 Department of Pathology, Tokai University Hachioji Hospital, Hachioji, Tokyo 192-0032, Japan

**Keywords:** gastric cancer, occult neoplastic cells, recurrence/metastasis, cytokeratin immnohistochemical staining, stage II/III

## Abstract

In the present study, we investigated the correlation between the presence of occult neoplastic cells (ONCs) in lymph node sinuses and recurrence/metastasis of stage II/III gastric cancer in 164 patients who underwent radical curative resection. We calculated the five-year relapse-free survival rate (5Y-RFS) and five-year overall survival rate (5Y-OS) of the ONC(+) and ONC(−) groups. The 5Y-RFS was 71.4% in the ONC(−) group and 47.5% in the ONC(+) group (P=0.003). The 5Y-OS was 68.8 and 48.4%, respectively (P=0.008). ONCs were found in 34.8% of stage II patients and were also detected in 66.7% of stage III patients. For distinguishing between the recurrence and non-recurrence groups, the sensitivity of ONC(+) was 64.5% (40/62; P=0.003), the positive predictive value (PPV) was 49.4% (40/81), the specificity was 59.8% (61/102) and the negative predictive value (NPV) was 73.5% (61/83). This high sensitivity indicates that ONC positivity may be a significant indicator for high-risk patients in the early postoperative period, and a lack of ONCs may be a useful indicator for identifying low-risk patients, as patients without ONCs had a high NPV.

## Introduction

The five-year survival rate of Japanese patients with stage II gastric cancer who receive curative resection is relatively good (68.3%). For stage III disease, however, survival is much lower at only 30.8% (1,2). In total, ~30% of stage II patients suffer from recurrence/metastasis after curative resection and this may be fatal (1,2). According to the pathology of breast cancer, positive lymph node metastasis indicates systemic disease with the potential for metastasis to other organs; therefore, the presence or absence of lymph node metastasis is considered to be one of the most important clinical markers for breast cancer patients (3,4). Recurrence, such as peritoneal dissemination, lymph node metastasis, or distant metastasis in patients with lymph node metastasis who undergo curative resection, is presumed to occur when cancer cells that enter the circulation during the perioperative period escape the immune system, enter the microcirculation of target organs and tissues, and find an appropriate microenvironment for growth and proliferation (5–7). Various studies have investigated the close correlation between tumor recurrence/metastasis and the detection of occult neoplastic cells (ONCs) that are positive for cytokeratin on immunohistochemical staining in the sinuses of lymph nodes distant from the primary tumor (8–13). Among patients showing recurrence of stage II/Dukes’ B colorectal cancer without lymph node metastasis, ~20–30% have ONCs. However, among patients showing recurrence of stage III/Dukes’ C colorectal cancer with lymph node metastasis, >70–80% have ONCs. This strongly suggests a correlation between ONCs and recurrence/metastasis (14,15). ONCs may be classified as single cells, clusters (2–10 cells forming a cluster <0.2 mm in diameter) and aggregates (>10 cells). It has been reported that freely floating ONC clusters in the lymph node sinuses are more dangerous for occult systemic metastasis than isolated tumor cells (≤0.2 mm) or micrometastases (0.2 to ≤2 mm) in the lymph nodes (14–16). Residual cancer cells in the microcirculation may be eradicated by early postoperative adjuvant chemotherapy, although tumor susceptibility to anticancer agents and the optimal dosage/administration schedule are factors that need to be considered. If it was possible to identify a high-risk group of patients with gastric cancer who are more likely to develop recurrence/metastasis, survival could be improved by providing potent adjuvant chemotherapy for these patients in the early postoperative period. In addition, identifying a low-risk group of patients who are not likely to develop recurrence/metastasis could contribute to reducing the psychological burden on these patients and to devising more appropriate follow-up schedules for them.

Cytokeratin is an epithelial marker that is useful for detecting micrometastases to lymph nodes, as >99% of normal nodes are not stained. AE1/AE3 and CAM 5.2 are anti-cytokeratin antibodies (17–21). Since the structure of cancer cells can be examined in detail, histological and immunohistochemical studies are superior to tests such as polymerase chain reaction in terms of assessing tumor cell viability and proliferative potential (17,18). Poorly differentiated adenocarcinoma and signet ring cell carcinoma as single cells without clusters are common histological types of gastric cancer, whereas colorectal cancer is usually well/moderately differentiated adenocarcinoma. Thus, ONCs are thought to have a different significance in gastric cancer from that of colorectal cancer with regard to morphological and biochemical characteristics, as the concept of clusters does not apply to gastric cancer, particularly in patients with poorly differentiated adenocarcinoma or signet ring cell carcinoma.

However, a detailed clinicopathological examination comparing the prevalence of ONCs with the clinical course, stage and/or tumor histological type has not been reported to date. Accordingly, the purpose of this study was to investigate the correlation between the presence of ONCs and the clinicopathological features of stage II/III gastric cancer by cytokeratin immunohistochemical staining of the lymph nodes in surgically resected specimens.

## Patients and methods

### Patients

We identified 186 patients with stage II/III gastric cancer from April 2005 to March 2012, who could be followed up to assess survival for more than five years on the basis of accurate and complete medical records. Among them, we investigated 164 patients (stage II, n=89; stage III, n=75) from whom >20 lymph nodes were collected. They included 62 patients (stage II, n=19; stage III, n=43) who had metastasis and/or recurrence within five years (recurrence group) and 102 patients (stage II, n=70; stage III, n=32) without metastasis or recurrence (non-recurrence group). We performed immunohistochemical staining for cytokeratin in the D2-dissected lymph nodes obtained from these two groups, and then compared the detection of ONCs with the clinical course. The study was approved by the ethics committee of Tokai University Hachioji Hospital (Tokyo, Japan). Informed consent was obtained from the patient.

### Methods

**Figure 1:**
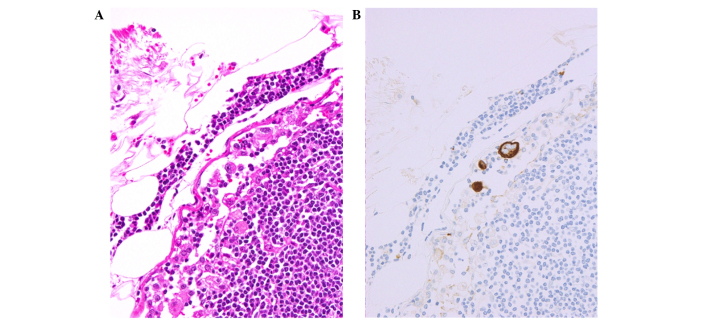
Occult neoplastic cells in lymph node sinuses detected by immunohistochemistry for cytokeratin and classified as floating single cells: (A) Hematoxylin and eosin staining and (B) cytokeratin staining. Magnification, ×400.

The routine indirect immunoperoxidase method was used for cytokeratin staining of the resected lymph nodes (17–19). Thin sections (3 μm) were prepared from the largest cut surface of each formalin-fixed and paraffin-embedded lymph node. After deparaffinization, the sections were immunostained using an automated staining apparatus (BenchMark^®^ XT; Roche Diagnostics K.K., Tokyo, Japan). After enzymatic treatment with protease 1 (0.5 U/ml; Roche Diagnostics K.K.) for 4 min at 37°C to activate the antigen, monoclonal anti-cytokeratin antibodies (AE1/AE3 and PCK26; Roche Diagnostics K.K.) were added as the primary antibodies, and the iVIEW DAB Detection kit (Roche Diagnostics K.K.) was employed for detection. Dehydration and mounting of the sections were performed after nuclear staining with hematoxylin. Hematoxylin and eosin staining and cytokeratin immunostaining were performed on serial sections of each lymph node in order to detect cancer cells. Tumor cells and/or tumor nests associated with fibrosis in the lymph nodes were not considered to be freely floating cells and were excluded from this study. Cytokeratin-immunostained cells freely floating in the lymph node sinuses (single cell type) were identified (Fig. 1) (5–7). We defined patients with ≥10 freely floating single cells as ONC(+) and patients with <10 freely floating single cells as ONC(−). Assessment was performed by S.Y., who was blinded to the clinical background of the patients; while M.M., Y.M. and K.K. performed data collection and analysis. The five-year relapse-free survival rate (5Y-RFS) and the five-year overall survival rate (5Y-OS) were calculated for the ONC(+) and ONC(−) groups. The incidence of ONC positivity in each stage (stages II or III) and histological type (signet ring cell, poorly differentiated or papillary/tubular type) was also calculated. Subsequently, the sensitivity, false positive (FP) rate, specificity, false negative (FN) rate, positive predictive value (PPV), negative predictive value (NPV) and predictive accuracy for distinguishing between the recurrence group and the non-recurrence group were determined. Additionally, the first site of recurrence was determined in ONC(+) patients from the recurrence group.

### Statistical analysis

The 5Y-RFS and 5Y-OS were calculated by the Kaplan-Meier method, while the log-rank test and hazard ratio (95% CI) were employed for comparison of survival between two groups. The χ^2^ test was used to compare the recurrence group with the non-recurrence group and risk ratios (95% CI) were calculated. P<0.05 was considered to indicate a statistically significant difference in all analyses. SPSS 17.0 statistical software (IBM SPSS, Statistics 17.0; International Business Machines Corp., Armonk, NY, USA) was used for these analyses.

## Results

**Figure 2:**
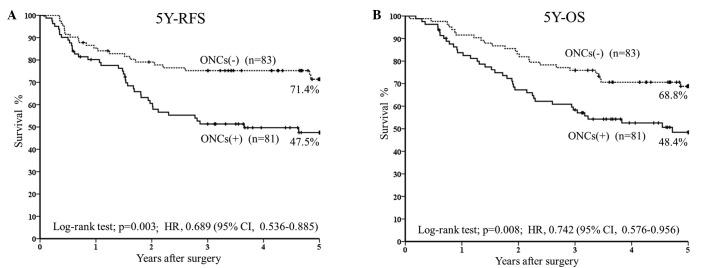
(A) Five-year relapse-free survival rate (5Y-RFS) and (B) 5-year overall survival rate (5Y-OS) calculated by the Kaplan-Meier method and compared with the log-rank test and hazard ratio (95% CI) between the recurrence and non-recurrence groups.

The 5Y-RFS was 71.4% (n=83) in the ONC(−) group and 47.5% (n=81) in the ONC(+) group (P=0.003; HR, 0.689; 95% CI, 0.536–0.885) (Fig. 2A), while 5Y-OS was 68.8% (n=83) and 48.4% (n=81), respectively (P=0.008; HR, 0.742; 95% CI, 0.576–0.956) (Fig. 2B).

Among stage II patients, 34.8% (31/89) were ONC(+), with the rate being 77.8% (7/9) in signet ring cell cancer, 51.9% (14/27) in poorly differentiated cancer and 25.0% (9/36) in papillary/tubular cancer (Table I). Among stage II patients, 65.2% (58/89) were ONC(−), with the rate being 22.2% (2/9) in signet ring cell carcinoma, 41.8% (13/27) in poorly differentiated adenocarcinoma and 75.0% (27/36) in papillary/tubular adenocarcinoma (Table I). Among stage III patients, 66.7% (50/75) were ONC(+), including 87.5% (7/8) with signet ring cell cancer, 76.2% (16/21) with poorly differentiated cancer and 59.5% (22/37) with papillary/tubular cancer (Table I). Among stage III patients, 33.3% (25/75) were ONC(−), including 12.5% (1/8) with signet ring cell cancer, 23.8% (5/21) with poorly differentiated cancer and 40.5% (15/37) with papillary/tubular cancer (Table I).

When ONCs were assessed in the 164 patients as a method of distinguishing between the recurrence/non-recurrence groups, the sensitivity of ONC positivity was 64.5% (40/62; P=0.003; HR, 0.689; 95% CI, 0.536–0.885), the FP rate was 40.2% (41/102), the specificity was 59.8% (61/102), the FN rate was 35.5% (22/62), the PPV was 49.4% (40/81), the NPV was 73.5% (61/83) and the predictive accuracy was 61.6% (101/164) (Table II).

In total, 40 out of 81 ONC(+) patients developed metastasis and/or recurrence within five years. The first site of recurrence was peritoneal dissemination in five cases (12.5%), local/lymph nodes in 19 cases (47.5%), the liver in six cases (15.0%), the lung in one case (2.5%), and others/unknown in nine cases (22.5%) (Table III).

## Discussion

In Europe and America, it is considered that D2 lymph node dissection during surgery on primary colon cancer contributes to the survival of stage II/Dukes’ B cases, but is not useful in the case of stage III/Dukes’ C patients (22). In Japan, the Guideline for Gastric Cancer Treatment recommends at least D2 lymph node dissection (1,2). The purpose of lymph node dissection during surgery is to achieve the complete en bloc removal of metastatic nodes, and it also contributes to standardizing the assessment of true node negativity by collecting a large number of lymph nodes with/without metastasis from specific regions (23). Identification of ≥12 lymph nodes is also recommended in the NCI guidelines for diagnosing true node negativity, and the most important prognostic factor is considered to be the number of metastatic foci observed in lymph nodes retrieved by D2 dissection (22–24). The JSCCR Guidelines for the Treatment of Colorectal Cancer include a detailed description of the criteria for high-risk cases who should receive postoperative adjuvant chemotherapy, which include lymph node metastasis affecting at least four nodes (TNM; N2), direct invasion of other organs, budding at the deepest leading edge of the primary tumor, and the presence of vascular involvement (25,26). The Japanese Guideline for Gastric Cancer Treatment recommends postoperative adjuvant chemotherapy for stage II/III patients, with the exception of those who are T1 cases, but does not provide a detailed description of the criteria for high-risk of recurrence/metastasis (1,2).

Colorectal cancer patients with lymph node metastases (stage IIIA or higher) are considered to have systemic disease, similar to breast cancer patients, and ~30–40% of them are presumed to be at high-risk of recurrence, while the remaining 60–70% belong to the low-risk group (14,15). In patients with stage III colorectal cancer, it is presumed that numerous free tumor cells have dispersed into the portal circulation and reached the liver, lungs and other organs, unlike the patients who have stage II/N0 localized tumors. The level of nonspecific immunity was also reported to be much lower in stage III patients than in stage II patients, which may be the result of persistent attempts by the immune system to eradicate ONCs (27,28).

Gastric cancers often initially recur as peritoneal dissemination or local lymph node metastasis, although one of the most common metastatic sites of colorectal cancer is the liver caused by venous invasion (29,30). It is thought that ONCs from gastric cancer enter small lymph vessels and then reach the peritoneal space. Initial recurrence as local lymph node metastasis is more frequent than that observed due to peritoneal dissemination, with first recurrence in the lymph nodes for 2/40 patients (5.0%) in N0, 7/40 patients (17.5%) in N1, 6/40 patients (5.0%) in N2, and 25/40 patients (62.5%) in N3 disease. As 77.5% of patients are N1 or N2, lymph node metastasis tends to affect the local nodes. However, more detailed clinicopathological examination of the characteristics of gastric cancer recurrence, such as peritoneal dissemination or local lymph node metastasis, is required.

In colorectal cancer, floating tumor cell clusters are considered to be the prime cause of distant metastasis to sites such as the liver or lungs (14,15). In the present study of gastric cancer, certain patients had relatively well-differentiated tumors and clusters of ONCs in their lymph nodes sinuses similar to those observed in colorectal cancer (data not shown). However, no significant difference was found between the recurrence group and the non-recurrence group based on comparison of clusters. The majority of colorectal cancers have the histology of well/moderately differentiated adenocarcinoma and poorly differentiated or special types are rare. By contrast, there are various histological types of gastric cancer, such as papillary adenocarcinoma, tubular adenocarcinoma, poorly differentiated adenocarcinoma and signet ring cell carcinoma (31). In this study, poorly differentiated (non-solid) and signet ring cell cancer, which are unlikely to form cell clusters, were frequent in the ONC(+) group, including 21/31 patients (67.7%) in stage II and 23/50 patients (46.0%) in stage III. It is thought that cells from poorly differentiated and signet ring cell cancers do not form clusters due to the abnormal expression of cell adhesion molecules (32,33). Floating single cells and clusters of well-differentiated adenocarcinoma form glandular structures as they proliferate, whereas poorly differentiated carcinoma cells are thought to persist as single cells or only form small nests. It is therefore suggested that the biological characteristics of gastric cancer mean that there is no correlation between floating clusters and recurrence/metastasis. The correlation between single tumor cells in the lymph node sinuses and recurrence/metastasis has not been clarified to date, possibly as only less viable ONCs become trapped in the lymph nodes and these cells are not involved in recurrence or metastasis (5–7,14,15). In patients who have <10 floating cells, ONC(−), these cells are assumed to be eradicated by the host immune system and are unlikely to cause recurrence or metastasis, whereas in those with ≥10 floating cells, ONC(+), certain cells may not be eliminated by the immune system and may proliferate to form micrometastases. Conventionally, tumors that show budding at the leading edge are thought to have a worse prognosis, but certain stage II/III gastric cancer patients without lymph node metastases show a relatively good prognosis (8–13). In the present study, ONC(+) stage II/III gastric cancer without lymph node metastasis was infrequent, accounting for 17.8% of cases (8/45). This finding suggests that ONC(−) stage II gastric cancer without lymph node metastasis is a localized tumor with a low risk of recurrence, similar to stage II/N0 colorectal cancer. In addition to factors such as host immunity and tumor susceptibility to anticancer agents, the results of this study suggest that ONC(+) may be a useful indicator for selecting patients with a high risk of recurrence in the early postoperative period. Conversely, the ONC(−) state is a useful indicator for identifying the low-risk group, as patients without ONCs exhibited a high NPV for recurrence. In the future, investigations should be performed to distinguish the high-risk group with a high sensitivity/high PPV and the low-risk group with a high specificity/high NPV. Clinical indicators with a high sensitivity/high PPV and a high specificity/high NPV should be identified to more accurately select the high-risk and low-risk groups for recurrence/metastasis, respectively.

## Figures and Tables

**Table I:** Clinicopathological features of stage II/III gastric cancer patients with each stage/histological type who were ONC(+) or ONC(−) in the lymph node sinuses.

Histological type	ONCs(+) 81 cases, n (%)	ONCs(−) 83 cases, n (%)	Total cases, n
Stage II (89 cases)			
Pap/tub	9 (25.0)	27 (75.0)	36
Poorly differentiated	14 (51.9)	13 (48.1)	27
Signet ring cell	7 (77.8)	2 (22.2)	9
Others/unknown	1 (5.9)	16 (94.1)	17
Stage III (75 cases)			
Pap/tub	22 (59.5)	15 (40.5)	37
Poorly differentiated	16 (76.2)	5 (23.8)	21
Signet ring cell	7 (87.5)	1 (12.5)	8
Others/unknown	5 (55.6)	4 (44.4)	9

ONC, occult neoplastic cell; pap, papillary type; tub, tubular type.

**Table II:** Detection rates of ONCs in the lymph node sinuses (positive, ≥10 floating single cells; negative, <10 floating single cells) in the recurrence and non-recurrence groups.

Total 164 cases (predictive accuracy 61.6%)	Recurrence group (n=62)	Non-recurrence group (n=102)
ONCs(+)	40 cases^a^	41 cases
81 cases (PPV 49.4%)	(Sensitivity 64.5%)	(FP rate 40.2%)
ONCs(−)	22 cases	61 cases
83 cases (NPV 73.5%)	(FN rate 35.5%)	(Specificity 59.8%)

a P=0.003, hazard ratio 0.689 (95% CI, 0.536–0.885).

ONCs, occult neoplastic cells; PPV, positive predictive value; NPV, negative predictive value; FP, false positive; FN, false negative.

**Table III:** ONC(+) patients in the recurrence group (n=40) and the patterns of recurrence/metastasis.

Site of reccurrence/metastasis	n (%)
Peritoneal dissemination	5 (12.5)
Local/lymph node	19 (47.5)
Liver	6 (15.0)
Lung	1 (2.5)
Others/unknown	9 (22.5)

ONC, occult neoplastic cell.

## Abbreviations

ONCs: occult neoplastic cells
5Y-RFS: five-year relapse-free survival
5Y-OS: five-year overall survival
PPV: positive predictive value
NPV: negative predictive value
FP: false positive
FN: false negative
